# Glucagon-like Peptide-1 Receptor Agonists in the Context of Eating Disorders: A Promising Therapeutic Option or a Double-Edged Sword?

**DOI:** 10.3390/jcm14093122

**Published:** 2025-04-30

**Authors:** Maria Kałas, Ewelina Stępniewska, Michał Gniedziejko, Jakub Leszczyński-Czeczatka, Mariusz Siemiński

**Affiliations:** 1 Department of Emergency Medicine, Medical University of Gdańsk, 80-214 Gdańsk, Poland; e.stepniewska@gumed.edu.pl (E.S.); mariusz.sieminski@gumed.edu.pl (M.S.); 2 Emergency Department, University Clinical Center, 80-952 Gdańsk, Polandjczeczatka@gmail.com (J.L.-C.)

**Keywords:** eating disorders, glucagon-like peptide-1 receptor agonists, binge-eating disorders

## Abstract

Glucagon-like peptide-1 Receptor Agonists (GLP-1 RAs) have been one of the most discussed issues in medicine for the past few years. Initially dedicated to patients with type 2 diabetes mellitus (T2DM), the medicine turned out to be an effective weight-loss treatment for people beyond this population. Whereas their beneficial somatic and metabolic effect are beyond doubt, their possible psychiatric adverse reactions have raised concerns. Eating disorders (EDs) are among the mental illnesses whose number is increasing worldwide. Thus, this review aims to summarize the status of knowledge on the correlation between the popularity of GLP-1 RAs and EDs. The conclusions are not unequivocal, pointing out that GLP-1 ARs have the potential to be an effective therapeutic option in some cases of Eds, but if used inappropriately, may increase morbidity of eating disorders.

## 1. Introduction

GLP-1 ARs have undoubtedly been one of the most discussed topics in medicine for the past few years. Even though they were initially dedicated to patients with T2DM, they have gained much popularity beyond this population as they turned out to be extremely effective weight loss medications [1]. Since obesity and T2DM are now worldwide plagues provoking many socio-economic consequences and leading to numerous comorbidities [2], it may seem that the Holy Grail has finally been found. There is no doubt about the positive influence of GLP-1 ARs on metabolic homeostasis as it has been well documented in numerous studies [1]. Moreover, the benefits of GLP-1 analogs seem to be much greater than initially expected. There is evidence that GLP-1 ARs may have favorable effect in the field of immunomodulation, neuroprotection, or cardiovascular health improvement. Data suggests that their use could be beneficial in case of neurodegenerative conditions, such as Alzheimer’s Disease or Parkinson’s Disease, as well as in some autoimmune diseases, e.g., rheumatoid arthritis [3]. GLP-1 analogs seem to be truly multipotential as they have even been studied for use in the treatment of Alcohol Use Disorder [4]. If ever adverse reactions of GLP-1 ARs are mentioned, the focus is mainly on the gastrointestinal side effects, including pancreatitis [5] or the possible elevated thyroid tumor risk [6]. However, lately there has been concern about the influence of long-term GLP-1 ARs therapy on mental health. Some data suggested that it may be harmful, leading to depression exacerbation or suicidal thoughts or attempts [7]. However, it was not confirmed in the research [7], and there was no definitive evidence of the causal relationship between the use of GLP-1 RAs in humans and the development of psychiatric adverse events [8]. Since eating disorders (EDs) are significant yet underestimated and are rising in number as a mental health issue worldwide [9], which increases mortality risk and contributes to reduced quality of life [10], the question that must inevitably arise is about the correlation between the popularity of GLP-1 ARs and the prevalence of EDs. Does the relatively easy and quick weight loss that they offer contribute to an increase in ED morbidity? On the other hand, since many patients diagnosed with ED suffer from obesity as well [11], are GLP-1 analogs a safe therapeutic option for them? Even though the efficacy of GLP-1 on energy balance control has been well documented, the data on the correlation between GLP-1 and eating disorders is relatively limited. However, some studies have shown a potential link between GLP-1 and binge-like feeding behavior, and existing preclinical data have shown changes in GLP-1 in animal models of binge eating [12]. Cognitive behavioral therapy (CBT) is an effective tool that helps to reduce the number of binge-eating episodes, but it is less efficient in terms of weight loss [13]. Thus, it seems that in case of disorders, such as binge eating, GLP-1 could potentially serve as a great support to previously used treatments. In this review, the authors aimed to address above questions and summarize the current knowledge on the topic of GLP-1 ARs and eating disorders.

## 2. Mechanism of Action and Current Indications for GLP-1 ARs

Glucagon-like peptide 1 is a hormone secreted predominantly by intestinal L cells, pancreatic alpha cells, and the central nervous system, which is indispensable to maintain glycemic homeostasis [14]. The artificial GLP-1 analog attaches to its receptor and activates the same signaling pathways as the intrinsic one [15]. As a result, the balance between the secretion of insulin and glucagon can be maintained, and the gastric motility and emptying slow down, which results in longer periods of satiety [16]. The advantage of analogs in comparison to the native human hormone is their longer half-life [17]. It has been 20 years since the first GLP-1 ARs (exenatide) was approved to treat T2DM in 2005 [1] and almost ten years later, in December 2014, the Food and Drug Administration approved liraglutide as a pharmacotherapy for obesity [18]. Although metformin remains the pharmacotherapy of the first choice for T2DM patients without any additional risk, according to the American Diabetes Association, GLP-1 analogs should be chosen as a first-line treatment, together with Sodium-glucose Cotransporters-2 (SGLT-2s), in case of T2DM and established chronic kidney disease (CKD), atherosclerotic cardiovascular disease (ASCVD), or indicators of high ASCVD risk independent of HbA1C levels [19]. As far as obesity is concerned, GLP-ARs are approved for patients with a Body Mass Index (BMI) > 30 or BMI > 27 and at least one weight-related complication, such as elevated blood pressure, hyperlipidemia, or DMT2, in addition to increased physical activity and reduced calorie diet [20]. The FDA-approved GLP-1 agonists are as follows: dulaglutide, semaglutide, liraglutide, exenatide, and tirzepatide (dual GLP-1 and GIP RAs). Lixisenatide and albiglutide were discontinued. A new agent, orforglipron, a nonpeptide oral GLP-1 receptor agonist, is under investigation [21]. In the past few years, there have been several studies comparing currently available GLP-1 ARs in terms of their efficacy and safety in different clinical contexts [22,23,24]. The findings confirm that GLP-1Ras, as a group, are generally safe and do not lead to hypoglycemia; however, agents differ in their efficacy in lowering HbA1c and reducing body weight [22]. In their meta-analysis, Xie et al. proved that the tirzepatide was the most effective GLP-1 agonist as far as the weight loss and lowering of HbA1c is concerned [22]; however, the oral route of administration, that is possible in case of semaglutide 14 mg, turned out to be more convenient for the patients and generally led to better compliance. The main disadvantage of this form of administration was an increased number of gastrointestinal adverse effects (nausea, diarrhea, vomiting) [22]. However, as previously mentioned, the enormous popularity of GLP-1 analogs attracted the interest of medical professionals from different specialties [25]. Psychiatry is not an exception. GLP-1 analogs have been investigated as a potential option of pharmacotherapy for the management of psychiatric disorders since obesity and diabetes have been considered as independent risk factors for mental illness since they may play significant role in pathogenesis of psychiatric disorders [26], e.g., patients with T2DM are twice as likely to develop depression in comparison to healthy population [27]. On the other hand, obesity and diabetes can also be consequences of psychiatric conditions and their treatment, with metabolic syndrome as a result of anti-psychotic treatment being the most obvious example [26]. Thus, enormous interest in GLP-1 led to various studies on its usefulness in psychiatry [26]. An investigation on GLP-1 analogs in the prevention of depressive episodes in patients with DT2 has been conducted [28] as well as their anti-psychotic potential or forementioned usefulness in Alzheimer’s Disease [29]. However, in this review, the authors would like to focus on a particular group of psychiatric patients diagnosed with eating disorders and GLP-1 ARs as a possible novel treatment option for them.

## 3. Eating Disorders Epidemiology

Eating disorders are a compound group of mental illnesses [30]. Their clinical picture is generally characterized by a disturbed attitude towards food, inappropriate eating behaviors, and disturbances in body image [31]. The nature of eating disorders is complex and multifactorial [32]. Both emotional difficulties and neurobiological factors have a role in the etiology of eating and weight disorders [33]. The correlation between genetic influences and environmental stressors also plays a significant role in the onset of ED [34]. The clinical spectrum of eating disorders is broad and includes both underweight, normal weight, and overweight patients [35]. Contrary to the popular belief that a person with eating disorders must be thin, only less than 6% of ED patients are medically underweight [36]. Some of the eating disorders, like anorexia nervosa and bulimia nervosa, are more connected with unrealistic body image and focus on achieving an ideal thin body. In order to achieve this goal, patients engage in various weight-control behaviors. Others, like binge-eating disorders (BEDs) and avoidant/restrictive food intake disorder (ARFID), do not include body weight and shape overevaluation as core diagnostic criteria and are mainly characterized by inappropriate eating behaviors [37]. The most recent studies confirm that the prevalence of eating disorders is high, especially in women [38]. The statistics on the incidence of ED vary; however, according to the Harvard Report [39], the overall lifetime prevalence may be as high as 8.60% among females and 4.07% among males. Moreover, data shows that the number of individuals diagnosed with eating disorders has been systematically rising, from 3.5% in 2000 to 7.8% in 2018 [38]. Previously, EDs were associated mainly with Western countries, but Makino et al. suggested that they are becoming more frequent also in non-Western countries [9]. What is even more alarming is the high prevalence of EDs among the young population. Disordered eating behaviors may affect over 22% of children and adolescents, with a predominance of girls [40].

According to the revised fifth version of the *Diagnostic and Statistical Manual of Mental Disorders* (DSM-5), five groups of eating disorders have been identified [41]:

- Anorexia nervosa (AN);
- Bulimia nervosa (BN);
- Binge-eating disorder (BED);
- Other specified feeding and eating disorder (OSFED);
- Avoidant/restrictive food intake disorder (ARFID).

Anorexia nervosa (AN) is a very common mental illness, the incidence rate of which is increasing in men and women. AN mainly affects teenagers and young women, and its occurrence reaches up to 4% in women during their lifetime, with an average time of illness of 6 years [42]. In case of AN prevalence in men is around 0.3% [43].

The complex of psychological, environmental, and genetic factors has an influence on the development of AN. Depression, anxiety disorders, drug abuse, or other comorbidities are the known psychological factors for AN prevalence [44].

Social media’s pressure about a thin posture [45], lack of support, and traumatic events are common environmental factors. Genetics also play an important role in AN development. It is confirmed that having a first-degree family member with AN can increase the risk of AN by 10 times [46].

The diagnostic criteria for AN according to the Diagnostic and Statistical Manual of Mental Disorders (DSM-5) are described below.

- Restriction of energy intake relative to requirements, leading to significantly low body weight for the patient’s age;
- Intense fear of gaining weight or persistent behavior that interferes with weight gain;
- Disturbance in the way in which one’s body weight or shape is experienced and undue influence of body weight or shape on self-evaluation [41].

Bulimia nervosa is the next common eating disorder according to the Diagnostic and Statistical Manual of Mental Disorders, which is characterized by binge eating followed by compensatory purging. There are many ways of engaging in purging behavior, which include vomiting induction, laxatives or diuretics abuse, fasting, and overexercising [47]. According to the DSM-5 criteria, the prevalence of BN in adult individuals is 4% to 6.7% [48] and affects both men and women, mainly in their youth, with prevalence in girls. A common factor that predisposes one to BN development is a psychiatric disease, such as affective disorders, anxiety, or drug abuse [49]. The diagnosis of BN is based on the three main elements:

- Binge eating episode—overeating within a 2 h period;
- Binge eating compensation—attempts at purging;
- Overfocus on body shape or weight.

Binge eating disorder (BED) is another common psychiatric condition that affects even 3% of the population. BED is a serious and complex psychiatric condition, where 30% of affected individuals are obese [41]. According to other studies, 50% of patients with obesity meet the criteria of BED [11], but eating disorders as an underlying cause of excessive weight gain may not always be identified [50].

Episodes of binge eating are characterized by a combination of excessive eating and a loss of control of consumed food that exceeds regular intake, which usually appears in 2 h intervals [51] when the individuals consume from 2000 kcal to 5000 kcal per episode [52]. BED, as well as other eating disorders, is usually caused by many psychological and environmental factors. Individuals suffering from eating disorders often experience distressing emotions. Media and societal pressures can influence perceptions of ideal body shape, contributing to feelings of inadequacy or stress, which might exacerbate symptoms of the disorder. Guilt is a distressing emotion that often accompanies ED and is a way to negotiate life difficulties [53]. It is commonly driven by moral thinking or dysfunction of self-control. Shame is also a common experience in the ED population [54]. After an episode of binge eating, patients suffer mentally and physically, but a binge-eating episode is not followed by compensatory behaviors, for example, purging, which is common in bulimia nervosa.

Anorexia nervosa, bulimia nervosa, and binge eating disorder are the three main groups of eating disorders due to the DSM-5 classification (Table 1). All other disorders that do not meet the criteria for any of the mentioned illnesses are classified into a new category of mixed eating disorders: “other specified feeding or eating disorder (OSFED)’’ or “unspecified feeding or eating disorder (UFED)” [55]. Atypical anorexia nervosa, subthreshold bulimia nervosa, and BED, among others, such as purging disorder (PD) or night eating syndrome (NES), are the eating disorders belonging to this group [56].

In situations where a patient does not meet all the criteria for an eating disorder or not all the information is completed, then the term UFED is applicable.

The last group of eating disorders, according to the DSM-5 classification, that needs to be mentioned is avoidant/restrictive food intake disorder (ARFID). This disorder is differentiated by avoidant or restrictive eating by its volume (decreased amount of food intake) or variety (avoidance of selected products). Nutritional deficits, decreased growth, weight loss, or the necessity of supplemented feeding are complications of this restricted diet [57]. Further research on the prevalence of ARFID in children and adults with autism, connected with to lack of interest in food, sensory-based avoidance, and phobia-based restriction, is conducted to continue with better treatment of this very specific group of patients [58].

## 4. Current Strategies in Eating Disorders Treatment

Treatment strategies differ between specific types of eating disorders. In some cases, therapy must be multidirectional because disturbed eating patterns turn out to be the tip of the iceberg, with underlying other mental problems like depression or anxiety that need to be addressed [59]. One must bear in mind that eating disorders also have medical comorbidities, some of them being very severe (e.g., cardiovascular, gastrointestinal, metabolic) [60], and they should be treated in parallel. Psychotherapy plays a major role in the therapy of each group of EDs as it has the most substantial impact on symptom reduction [37] and helps to disentangle the underlying emotional and behavioral causes [61]. Various psychological approaches are being used, e.g., cognitive behavioral therapy, family therapy, or more structured, specialized treatments, such as the Maudsley model of anorexia treatment for adults (MANTRA). Cognitive behavioral therapy is a therapy based on resolving present difficulties in a patient’s life as well as changing damaging patterns of action. CBT has an increasing role in the treatment of ED in young patients, but treatment of AN is still a challenge [62]. On the other hand, metacognitive therapy is based on the revelation of the patient’s thoughts, patterns of thinking, later exposition of their influence on the decisions or feelings of the patient, and finally finding an alternative way to react and decrease the symptoms. MCT is found to be beneficial in AN [63] but requires further analysis.

As far as pharmacotherapy is concerned, it mainly has an adjunctive function [64]. Lisdexamfetamine, a pro-stimulant initially registered to treat attention deficit hyperactivity disorder (ADHD) [59], is the only FDA-approved pharmacotherapy for BED, and although it decreases binge eating episodes, its use can be limited by adverse effects [65]. What is unfavorable in the case of ED patients, since eating disorders and substance use disorders often co-occur [66], as a stimulant, lisdexamfetamine poses the potential for abuse [67]. An anticonvulsant, topiramate, is sometimes used off-label in BED treatment, but its use is limited by numerous contraindications [68]. In the case of AN, there is no pharmacological treatment with proven efficacy [69]. For BN, the only FDA-approved pharmacological therapy is fluoxetine [65], which belongs to the selective serotonin reuptake inhibitors (SSRI) group of antidepressants [70]. It is effective at reducing both binge eating and vomiting episodes, but it is not free from adverse effects. Topiramate, an antiepileptic drug, is sometimes used off-label for BED, and while effective, its use may also be limited due to many side effects and drug interactions [65]. Unfortunately, the efficacy of current methods of ED treatment is not satisfactory, and the failure rate may be up to 30% [64] and the rates of chronicity are high [69].

## 5. GLP-1 ARs and Their Possible Use a Part of Therapeutic Strategy in EDs

In order to achieve progress in pharmacotherapeutic strategies to treat eating disorders, it is crucial to understand the underlying neurobiological mechanisms [12]. Studies based on animal models have shown the correlation between the dysregulation of the endogenous GLP-1 system and binge-eating behaviors [65]. In Japanese quails, GLP-1 administered via intracerebroventricular injection led to reduced food intake. The c-Fos immunoreactivity was quantified at 60 min post-injection in hypothalamic and brainstem nuclei that mediate food intake and determined that the hypothalamic paraventricular nucleus, nucleus of the solitary tract, and area postrema of the brainstem were activated in response to GLP-1. These results suggest that GLP-1 induces anorexigenic effects in the central nervous system that are likely mediated at the level of the hypothalamic paraventricular nucleus and brainstem [71]. Initial human data indicated blunted postprandial GLP-1 release in patients with BN and BED [72]. The influence of GLP-1 ARs on food intake by stimulating satiety center in the brain has been well documented [73]. It is achieved by activating GLP-1 receptors, which reduce food intake, body weight, and stimulate insulin secretion in a glucose-dependent manner [61,74]. However, GLP-1 ARs should be considered as a therapeutic option also in the context of comorbidities of BED since the beneficial influence of GLP-1 agonists on metabolic health, impairment of which is a common concern in BED patients, has also been demonstrated [75]. Moreover, from the point of view of patients suffering from BED, among whom the prevalence of food addiction is higher than in other eating disorders except in bulimia nervosa [76] an important effect of GLP-1 ARs is their action on reward-driven feeding by activating hypothalamic and hindbrain areas involved in feeding control, reward pathways like the ventral hippocampus, and forebrain regions like the medial prefrontal cortex [61]. Since the two main goals of the BED therapy are body weight reduction and the cessation of binges [77], it seems to be a natural consequence that the potential of GLP-1 analogs as a therapeutic tool in a group of BED has been investigated. The existing literature on this topic is not extensive, yet it is encouraging. According to the current data, the potential of GLP-1 analogs as pharmacological treatments in cases of BED seems to be substantial. Notably, apart from favorable metabolic effects, as well as reduction in binge-eating behaviors, one of the remarkable advantages of GLP-1 ARs in context of binge-eating disorders treatment, encouraging further studies on this topic, is their proved favorable psychiatric side effect profile compared to existing pharmacological options [61]. On the other hand, an animal-based study, in which the GLP-1R antagonist exendin-9 (Ex-9) was delivered into the fourth ventricle of hepatoma tumor-bearing rats, showed that it attenuated the cancer anorexia-cachexia syndrome. In the same study, it was demonstrated that hepatoma tumor-bearing animals with a knockdown of GLP-1 expression in the nucleus tractus solitarii had higher food intake and reduced body weight loss compared to the tumor-bearing control group [78]. Even though the cancer anorexia-cachexia syndrome can not be extrapolated to the anorexia nervosa population, the concept of using GLP-1 antagonist in restrictive eating disorders therapy seems worth further investigation.

### 5.1. Current Studies Using GLP-1 ARs in Binge-Eating Disorder Treatment

Since the disruption of GLP-1 [65] was proved to be one of the underlying causes of BED, and it is implicated in the hypothesis of the possible use of GLP-1 agonists as a part of the therapeutic strategy [79]. Thus, parallel with the registration of GLP-1ARs as weight-loss medications, the studies on their possible use in BED treatment were initiated. Ten years ago, Robert et al. [80] published the result of their study on the effects of liraglutide on appetite and plasma ghrelin in non-diabetic obese participants with subclinical binge-eating. Findings revealed that participants treated with liraglutide in addition to standard exercise and dietary procedures showed a significant decrease in binge eating episodes, together with a reduction in body weight, BMI, waist circumference, systolic blood pressure, and improvement of metabolic profile in comparison to the control group. However, it was noticed that ghrelin levels were significantly increased, which may potentially diminish the weight loss effects of liraglutide beyond the intervention. Another study by Da Porto et al. [81] evaluated the efficacy of dulaglutide vs. gliclazide in the reduction of binge-eating episodes, body weight, BMI, percentage fat mass, and HbA1c in type 2 diabetic patients with binge eating disorder. In this study, the results were also in favor of GLP-1 analogs, which turned out to be more effective.

In 2022r. Allison et al. [67] published the results of their pilot randomized controlled trial of liraglutide 3.0 mg for binge-eating disorders. The investigated group was small (27 participants), yet the results were promising. The liraglutide group had a significantly larger mean weight loss of 4.7 kg compared to the 0.9 kg loss of placebo-treated participants. As far as binge episodes are concerned, there were no significant differences between the treatment groups in the percentage of participants who achieved binge-eating remission. Richards et al. investigated the effects of another GLP1-AR, semaglutide, on Binge Eating Scale (BES) scores in individuals with BED in comparison to previously used substances [68]. Patients were divided into three groups: those prescribed semaglutide, those prescribed either lisdexamphetamine or topiramate, and those prescribed a combination of semaglutide with lisdexamphetamine or topiramate. The results revealed that the patients receiving semaglutide only achieved greater reductions in BES scores compared to the other groups. Combined pharmacotherapy with both semaglutide and the other anti-obesity medications did not result in greater reductions in BES scores compared to the semaglutide-only group. The outcome of the study seems very promising; however, a serious limitation to this study was the small number of participants, as the study was inclusive of only 48 individuals [68].

To date, the longest study on GLP-1 ARs in eating disorders with the greatest number of participants was conducted by Chao et al. [82]. In this study, 150 individuals with obesity were randomly divided into behavioral weight loss treatment (BWL), liraglutide + BWL and liraglutide, and BWL and a low-calorie portion-controlled diet.

Participants were assessed after 24 and 52 weeks. The short-term results were in favor of liraglutide + BWL and liragludite + BWL + diet groups, but attenuated in time.

The most recent publication on the topic of BED treatment using GLP was released in February 2025 [83]. Radkhah et al. published the first systematic review and meta-analysis on the efficacy of GLP-1 analogs in the treatment of eating disorders [83]. In this publication, they confirmed the efficacy of GLP-1 in managing BED, revealing that patients receiving GLP-1 agonists experienced greater weight loss compared to controls. GLP-1 agonists significantly reduced BMI and waist circumference. Binge Eating Scale scores improved significantly, though heterogeneity was noted. Once again, the limitation is the size of the study group. Radkhah et al. included five studies in their meta-analysis, with a total of 182 patients [83].

### 5.2. The Future Perspectives and Safety of Combining GLP-1 ARs with Currently Used Pharmacological Agents for Eating Disorders

As far as interactions between GLP-ARs and standard used therapeutic agents for eating disorders are concerned, data are limited. However, it seems that in the future, combined therapies may turn out to be beneficial because, according to the study by Ripken et al., GLP-1 release from intestinal ileal tissue following stimulation by different (non-)nutritional stimuli could be further increased by adding the SSRI fluoxetine [84]. Thus, comparative studies involving large groups of BED patients treated with GLP-1 ARs and fluoxetine versus only GLP-1 analogs/fluoxetine would be favorable. As far as the interactions between lisdexamfetamine and GLP-1 analogs are concerned, apart from the study by Allison et al. [67], there is no research on this subject. Although in this study, no remarkable adverse drug interactions were observed.

## 6. GLP-1 ARs: Can They Increase the Prevalence of Eating Disorders?

Eating disorders are a broad spectrum of dysregulated eating behaviors, from previously discussed binge-eating disorders at one end of the scale to over-controlled eating patterns like anorexia nervosa at another. The subject of drug misuse and abuse for weight-loss purposes is not new and has already been reported in the literature [85]. However, the public interest in GLP-1 ARs therapy that has been growing enormously for the past few years [86] brings new risk. Social media promotes the use of GLP-1 ARs for esthetic purposes [86], presenting them as a miraculous weight-loss treatment [87]. The powerful influence of celebrities and social media on current beauty trends has been demonstrated in various studies [88]. Thus, the question that arises is about the possibility of another plague of EDs, especially those restrictive ones, as a result of GLP-1 ARs (mis)use. Is there a threat that increased and longer satiety, which is advantageous in case of BED treatment or proper weight-loss therapy, may provoke restrictive eating behaviors in some patients, especially those who take GLP-1 ARs without actual medical supervision? Another dangerous aspect of GLP-1 analog therapy in the context of eating disorders is the possibility of perpetuation of inappropriate eating habits. Since GLP-1 ARs promote satiety, is there a risk that they may potentially lead to some nutritional deficits in individuals prioritizing palatability over nutritive value of alimentation, especially if weight-loss therapy is not holistic and focused on lifestyle changes? Whereas studies on possible adverse psychiatric effects of GLP-1 ARs, like increased suicidal risk, have already been conducted and such causal relationship has not been confirmed [8], the subject of potential unfavorable impact of GLP-1 analogs on developing or persisting eating disorders has not been well documented yet. Thus, according to the current literature, the above questions cannot be answered satisfactorily. However, the topic of inappropriate use of GLP-1 ARs is starting to appear in public debate as it affects all the age groups. GLP-1 analogs have also been approved in pediatric patients and [89], unfortunately, examples of the misuse of GLP-1 ARs in this population have been described so far [90]. This is very alarming since eating disorders have a peak in adolescence [91] and are also triggered by social media [92], of which young people are active users. In 2023, Chiappini et al. [85] published the outcome of the first study on the misuse and abuse potential of semaglutide in comparison with both remaining GLP-1 analogs and the phentermine–topiramate combination. The results revealed that semaglutide was associated with remarkably higher levels of abuse, intentional product use issues, and use without a prescription [85]. Further studies on this topic need to be conducted; however, these findings are interesting in the context of the safety of individual GLP-1 analogs. A valuable perspective on the issue of the potential exacerbation of EDs due the (mis)use of GLP-1 Ars, from a practical point of view, was presented by Bartel et al. [93]. The authors base this research on their own experience since they have come across a problematic use of GLP-1 analogs. Thus, they proposed a guide for ED physicians on how to discuss GLP-1 ARs in clinical practice. They also made valuable suggestions concerning future research. The authors suggested that eventual studies should include longer term follow-ups (up to 5 years) because ED symptoms could have a delayed onset due to prolonged medication effects over time and symptom rebound with medication discontinuation. Bartel et al. also postulated that further research in ED populations should not only concentrate on injectable forms of GLP-1 analogs but also examine the misuse of the oral forms of GLP-1 ARs as they become more popular and available. Finally, they hope that in the future the identification of patients with high risk of ED due to GLP-1 ARs will be facilitated by artificial intelligence algorithms [93].

## 7. Conclusions

The results of the presented studies on the use of GLP-1 ARs in BED treatment are promising, yet they should be considered with caution. Firstly, only one study by Allison et al. [67] was a blinded randomized control trial comparing GLP-1 ARs versus placebo. Other studies were open-label trials and assessed the efficacy of GLP-1 analogs in comparison to standard therapeutic strategies in BED. Secondly, probably due to a small number of participants, the studies lack heterogeneity. The most diverse population took part in the studies by Richards et al. and Chao et al. [68,82], which were inclusive of minorities and the Hispanic/Latino population. The small diversity of the study participants in terms of race, culture, and socio-economic environment influences the generalizability of the findings. Another factor that should be taken into consideration is the fact that some studies excluded patients with somatic or psychiatric comorbidities, e.g., participants could not have more than moderate depressive symptoms or recent suicidality (Allison et al. [67]) or T2DM (Chao et al. [82]), whereas patients with BED often struggle with other mental and metabolic problems and it seems to be reasonable to include such population in future studies. Finally, the groups treated with GLP-1ARs presented a reduction in the number of binge-eating episodes, but the difference between groups was not statistically significant (Allison et al., Chao et al. [67,82]). However, the outcome that was undoubtedly achieved was greater body weight reduction among patients treated with GLP-1ARs. It leads to a conclusion that GLP-1 agonists may be valuable additional therapeutic option for BED patients but should not be regarded as a sole remedy since it is indispensable to address the emotional causes of eating disorders as there is evidence that individuals suffering from BED have cognitive deficits relating to uncertainty, inhibition, consistency, and flexibility [94].

## 8. Discussion

GLP-1 ARs are a relatively new group of weight loss medications so the current state of knowledge on their influence on ED is limited. According to the existing data, GLP-ARs may potentially play a significant role in the therapy of patients with BED. However, the available data are based on trials with only a small number of participants. Since the prevalence of ED is rising and the present therapeutic options are not extremely effective, efforts to develop alternative treatment methods would be worthwhile. Considering the complex etiology of BED, the GLP-1 ARs will probably not be the sole remedy to this problem, but seems to be a promising pharmacological option and worth further investigation as an additional treatment to psychotherapy in patients with this specific type of ED. Therefore, future studies should be based on more diverse populations and should also include populations with other psychiatric and somatic comorbidities. On the other hand, it seems that so far, the psychiatric adverse effects of GLP-1 ARs have been underestimated and more attention should be paid to the regular mental health assessment of patients treated with GLP-1 analogs. Physicians, especially obesity specialists, need to be aware of the fact that some of their patients may develop mental disorders during GLP-1 analog treatment, including an inappropriate attitude towards food. Attention should be paid to any symptoms of GLP-1 misuse in the course of therapy. Therefore, an evaluation of patient’s psychological status before and during GLP-1 ARs treatment would seem to be beneficial. It would help to identify a group of patients most vulnerable to develop ED in course of treatment with GLP-1 ARs and think of an alternative therapeutic option for them.

## 9. Limitations

The authors are aware that this review has several limitations. Firstly, the presented studies suggesting the beneficial role of GLP-1 analogs in BED pharmacotherapy are not numerous, and they are based on small groups. Moreover, the diversity among participants, including minorities, was present in just one of them. Secondly, the sole study was a randomized control trial, and the overall duration of the study was relatively short, which does not allow long-term predictions. Finally, the hypothesis of GLP-1 analogs as a trigger of restrictive eating disorders is not well established in the literature and needs further verification in long-term studies that should be more holistic and concentrate not only on the physiological effects of GLP-1 RAs therapy such as anthropometric measurements but also on the psychological impact they have on patients.

## Figures and Tables

**Table 1:** In the table below is a comparison of the three main types of eating disorders.

	Anorexia Nervosa (AN)	Bulimia Nervosa (BN)	Binge Eating Disorders (BED)
Key characteristics	Intense fear of gaining weight, distorted body image, and extreme restriction of food intake	Recurrent binge eating followed by compensatory behaviors (e.g., vomiting)	Recurrent episodes of eating large amounts of food without purging
Eating behavior	Very restrictive and low-calorie food intake	Binge episodes, loss of control, overeating, followed by guilt.	Cycles of restrictive and overeating cycles, loss of control while eating
Purging	Overexercise, self-induced vomiting, and laxative use are also common	Self-induced vomiting, use of laxatives or diuretics, over-exercise, or restrictive eating after binges	Purging is not common
Body image	Extreme concern with body shape and weight, distorted view of body image, fear of being fat	Extreme concern with body shape and weight	Extreme concern with body shape and weight
Body weight	low body weight, less than 85% of expected body weight	Maintained at or above a minimally normal level	Usually overweight
Risk factors	Perfectionism, trauma, and societal pressure	Low self-esteem, body dissatisfaction, and stress	Depression, anxiety, history of dieting

## Abbreviations

The following abbreviations are used in this manuscript:

ADHD	Attention deficit hyperactivity disorder
AN	Anorexia nervosa
ARFID	Avoidant/restrictive food intake disorder
ASCVD	atherosclerotic cardiovascular disease
BED	Binge-eating disorders
BN	Bulimia nervosa
ED	Eating disorders
FDA	U.S. Food and Drug Administration
GLP-1 ARs	Glucagon-like peptide-1 Receptor Agonists
HbA1c	Hemoglobin A1c
OSFED	Other specified feeding and eating disorder
SGLT-2	Sodium-glucose Cotransporter-2
T2DM	Diabetes mellitus type 2
